# Norcantharidin promotes M1 macrophage polarization and suppresses colorectal cancer growth

**DOI:** 10.1038/s41401-025-01578-8

**Published:** 2025-05-20

**Authors:** Xiao-man Wei, Si-cheng Lu, Liu Li, Ying-jie Gao, Jun-yi Wang, Song-yang Xi, Ling-yu Linda Ye, Wei-xing Shen, Mian-hua Wu, Dayue Darrel Duan, Hai-bo Cheng

**Affiliations:** 1 The First Clinical Medical College of Nanjing University of Chinese Medicine, Nanjing, 210023 China; 2 Jiangsu Collaborative Innovation Center of Traditional Chinese Medicine in Prevention and Treatment of Tumor, Nanjing, 210023 China; 3 School of Integrative Medicine of Nanjing University of Chinese Medicine, Nanjing, 210023 China; 4 Affiliated Hospital of Nanjing University of Chinese Medicine, Nanjing, 210029 China; 5 Department of Pharmacology, University of Nevada Reno School of Medicine, Reno, NV 89557 USA

**Keywords:** colorectal cancer, norcantharidin, tumor microenvironment, M1 macrophage, CSF2, JAK2/STAT3 pathway

## Abstract

Colorectal cancer (CRC) is characterized by an immunosuppressive and inflammatory microenvironment, thus responds poorly to therapy. Previous studies show that norcantharidin (NCTD), a demethylated cantharidin (CTD) derived from *Mylabris*, exerts high efficacy in treating various cancers. In this study we investigated the antitumor effects of NCTD against CRC and the underlying mechanisms. Subcutaneous CRC models were established in *balb/c* mice using mouse colorectal cancer cell line CT26 and in *balb/c* nude mice using human colorectal cancer cell line HCT116. The mice were administered NCTD (2 or 4 mg·kg^−1^·d^−1^, i.p.) for 14 days. We showed that NCTD dose-dependently reduced the tumor growth in both the CRC models. Furthermore, NCTD markedly increased M1 macrophage infiltration in tumor tissue in both the CRC models. NCTD-induced macrophage M1 polarization was confirmed by flow cytometry and qPCR assays in both THP-1 cell-derived and RAW264.7 macrophage models in vitro. We demonstrated that NCTD (20, 40 μM) dose-dependently increased CSF2 secretion from CRC cells and macrophages, and suppressed the JAK2/STAT3 signaling pathway in CRC cells. Concurrently, NCTD (10–40 μM) dose-dependently inhibited CRC cell proliferation, invasion and migration in vitro. In conclusion, this study provides new evidence for the effects of NCTD against CRC and elucidates its antitumor mechanisms through remodeling the inflammatory microenvironment via CSF2-mediated macrophage M1 polarization and inhibiting JAK2/STAT3 phosphorylation in CRC cells.

## Introduction

Colorectal cancer (CRC) remains one of the leading causes of morbidity and mortality worldwide, seriously affecting human health and life expectancy [1, 2]. The immunosuppressive and inflammatory microenvironment poses a major barrier to effective antitumor therapy in CRC patients [3, 4]. To date, chemotherapy and immunotherapy for CRC remains unsatisfactory due to low efficacy and high adverse effects [5]. Therefore, there is an urgent need to explore better agents for CRC therapy.

Macrophages are pivotal components of the tumor microenvironment (TME), influencing the activities of other immune cells. They can be categorized into two subsets: classical M1 macrophages and alternative M2 macrophages [6, 7]. M1 macrophages predominantly secrete pro-inflammatory molecules and play a role in presenting tumor-specific antigens to T cells and supporting antitumor immunity. The M1 subtype of macrophages, when activated in a classical manner, has been shown to kill and move tumor cells by the generated NO, ROS [8, 9]. Several studies have shown the colony-stimulating factor 2 (CSF2), also known as granulocyte-macrophage colony-stimulating factor (GM-CSF) plays a key role in the polarization of macrophages and enhances their pro-inflammatory capabilities to attack tumor cells and stimulate adaptive immune responses [10–12]. The Janus kinase 2 (JAK2) / signal transducer and activator of transcription 3 (STAT3) signaling pathway has been associated with immune response, inflammation and cell proliferation [13]. Recent study has suggested that the inhibition of the STAT3 pathway can stimulate M1 macrophage polarization primarily through inhibition of STAT3 which decreases the expression of anti-inflammatory genes, thus allowing the pro-inflammatory pathway to dominate [14].

**Fig. 1:**
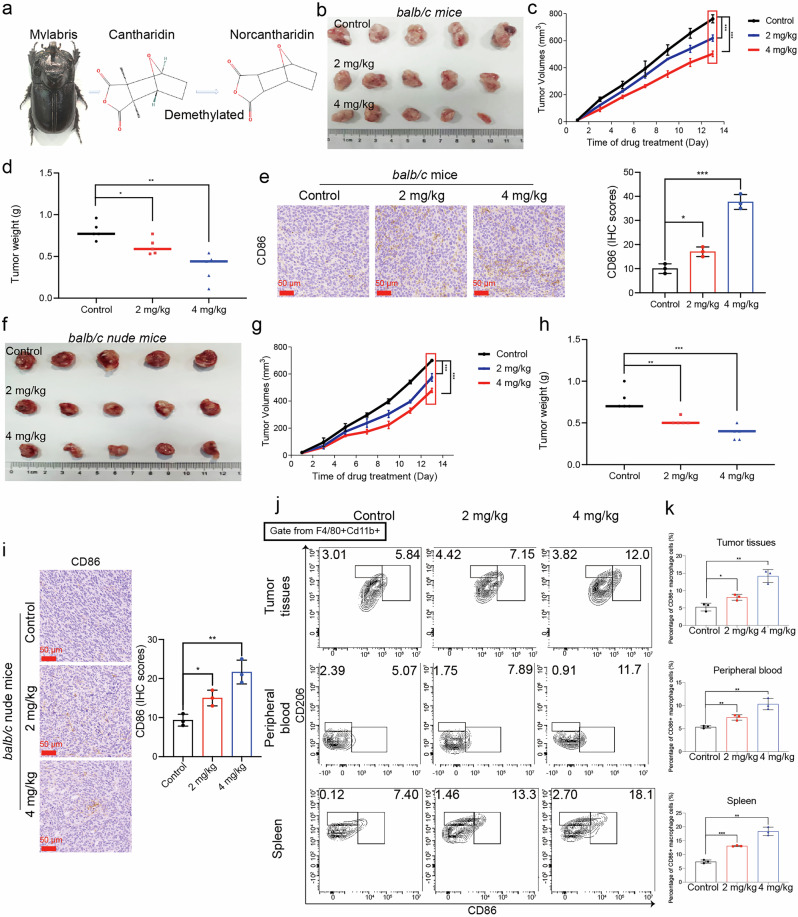
Antitumor activity of NCTD in vivo. **a** Chemical structure of NCTD. **b** Representative tumor photographs from CT26 tumor-bearing mice treated with NCTD and control. **c** Curves of changes in tumor volume in each group (*n* = 5). **d** Weight of each group of subcutaneous tumors (*n* = 5). **e** Left: Representative CD86 immunohistochemistry images of each group of tumors (200× magnification, scale bar: 50 μm). Right: Statistical histograms of the IHC scores (*n* = 3). **f** Representative tumor photographs from HCT116 tumor-bearing mice treated with NCTD and control. **g** Curves of changes in tumor volume in each group (*n* = 5). **h** Weight of each group of subcutaneous tumors (*n* = 5). **i** Left: Representative CD86 immunohistochemistry images of each group of tumors (200× magnification, scale bar: 50 μm). Right: Statistical histograms of the IHC scores (*n* = 3). **j** Results of flow cytometry analysis to detect the proportion changes of M1 and M2 macrophages in tumor tissues, peripheral blood and spleen of mice. **k** Statistical analysis of the percentages of CD86^+^ macrophage cells (*n* = 3). Data are presented as mean ± SD. *P < 0.05, **P < 0.01, ***P < 0.001.

Numerous studies have reported that norcantharidin (NCTD), a demethylated cantharidin (CTD) derived from *Mylabris* (Fig. 1a), is a potent anti-cancer compound that is more effective and less toxic than CTD [15]. In addition, it has been known that NCTD also possesses anti-inflammatory properties in various inflammation-related disease models [16–18]. In hepatocellular carcinomas, NCTD induced macrophage polarization by augmenting miR-214 [19]. However, the role of NCTD in CRC remains unknown.

In the present study, we demonstrated that NCTD exhibits antitumor activity in CRC cells by inducing apoptosis through the JAK2/STAT3 signaling pathway. This disruption results in reduced intracellular reactive oxygen species (ROS) and cell cycle arrest. Meanwhile, it is found that NCTD increased both mRNA and protein levels of CSF2 in CRC and macrophage cells, which promotes the M1 macrophage polarization in vivo and in vitro at whole animal and molecular levels.

## Materials and methods

### Animals models

The subcutaneous CRC models were used to evaluate the antitumor effect of NCTD in vivo. A total of 2 × 10^6^ CT26 cells in 100 μL PBS were subcutaneously injected into the dorsal flank of *balb/c* mice (*n* = 5 for each group). In addition, 2×10^6^ HCT116 cells were subcutaneously injected into the dorsal flank of *balb/c* nude mice (*n* = 5 for each group). Mice were randomized into Control group (PBS, once a day, i.p.), NCTD 2 mg/kg group (i.p., once a day), and NCTD 4 mg/kg group (i.p., once a day). Tumor volume was monitored after inoculation according to the following formula: volume = length × width^2^ × 0.5.

The macrophage clearance mouse model was constructed to evaluate whether the antitumor effects of NCTD are dependent on macrophages. Clodronate liposomes were employed for the depletion of macrophages in mice. The clodronate liposomes (LipoCLO, #40337ES10, Shanghai, China) and the control PBS liposomes (LipoPBS, #40338ES10, Shanghai, China) were procured from Yeasen. One day prior to the injection of cancer cells, 100 µL of LipoCLO (at a concentration of 5 mg/mL) or LipoPBS was administered intraperitoneally. This liposomal administration was subsequently repeated every two days.

This investigation conforms to the Guide for the Care and Use of Laboratory Animals (US National Institute of Health, publication No. 85–23, revised 1996) and was in accordance with the institutional guidelines for animal care and use approved by the Institutional Animal Care and Use Committee of Nanjing University of Chinese Medicine.

The present study obtained the endorsement of the Ethics Committee for Animal Experimentation of Nanjing University of Chinese Medicine and was conducted in strict compliance with the regulations set forth by the committee.

### Cell lines and culture

Human colorectal cancer cell lines HCT116 and LoVo, mouse colorectal cancer cell lines CT26, the human monocyte cell line THP-1 and the mouse macrophage cell line RAW264.7 were obtained from the Cell Bank of China Science Academy (Shanghai, China). HCT116, CT26 and THP-1 cells were cultured in RPMI-1640 medium, LoVo cells were cultured in F-12K medium and RAW264.7 cells were cultured in DMEM medium (Basalmedia Technologies, China). All medium was supplemented with 10% fetal bovine serum (ABWBIO, China), 1% penicillin-streptomycin (Basalmedia Technologies, China). All cells were cultured in a humidified atmosphere containing 5% CO_2_ at 37 °C.

### Macrophage polarization

Human monocytic THP-1 cells were differentiated into M0 macrophages by treatment with 200 ng/mL Phorbol 12-myristate 13-acetate (PMA, MedChemExpress, #HY-18739) for 48 h. To generate M1-polarized THP-1 macrophages, THP-1 cells were incubated with PMA (200 ng/mL) for 24 h and then treated with 100 ng/mL PMA plus 100 ng/mL lipopolysaccharide (LPS, MedChemExpress, #HY-D1056) and 20 ng/mL recombinant human IFN-γ (Proteintech, #HZ-1301) for a further 48 h. To generate M1-polarized RAW264.7 macrophages, RAW264.7 cells were stimulated with 100 ng/mL LPS plus 20 ng/mL recombinant mouse IFN-γ (MedChemExpress, #HY-P7071) for 24 h.

### Cell viability assay

Cell Counting Kit-8 (#40203ES60, Yeasen) was used to evaluate cell viability as described previously [20]. Briefly, 8 × 10^3^ cells were planted into 96-well plates, after adhesion, cells were incubated with different dose of NCTD for 48 h or 24 h. The plates were added with CCK‑8 solution (10 μL/well) at a specific time and incubated for another 2 h at 37 °C. Cell viability was assessed by optical density measurements.

### Colony formation assay

The cells were seeded in 12-well plates (800 per cell) with different doses of NCTD. When the cells showed obvious colonies after 14 days, colonies formed by at least 50 cells were stained with crystal violet and counted.

### Ethynyl-2′-deoxyuridine (EdU) staining

EdU cell proliferation staining was performed with an EdU staining kit (Beyotime, #C0071S) following the manufacturer’s protocol. Briefly, 2 × 10^5^ CRC cells were seeded in 12-well plates and treated with NCTD for 48 h. Subsequently, EdU solution (10 µM) was added to the cell medium for 6 h, followed by fixation, penetration and three washes of the cells. The click reaction solution containing Azide 488 was then applied to the cells for 30 min and cell nuclei were counterstained with Hoechst 33342 buffer.

### Apoptosis assay

Apoptosis was measured using the Apoptosis Detection Kit (Yeasen, #40304ES50) as described previously [10]. Briefly, cells were plated in a 6-well plate with 1 × 10^6^ cells per well concentration and treated with various concentrations of NCTD. After 48 h, cells were washed twice with PBS and incubated with Annexin V-Alexa Fluor 647/PI for 15 min at room temperature.

### Cell-cycle assay

Cells were fixed with ice-cold 70% ethanol at −20 °C for overnight. The fixed cells were washed with ice-cold PBS, incubated with RNase A (10 mg/mL, Solarbio Biological Technology, #R1030) in PBS at 37 °C for 1 h, and then stained with 10 μg/mL propidium iodide (PI, Yeasen, #40711ES10) solution.

### Analysis of different T and macrophage cells detected by flow cytometry

Peripheral blood mononuclear cells (PBMCs) were collected and isolated using the mouse peripheral blood lymphocyte isolation kit (Solaibao Biological Technology, #P8620). The mouse spleens were harvested and grinded in 70 μM cell strainer to obtain a single-cell suspension, subsequently the cell suspensions were enriched and purified with a mouse spleen lymphocyte isolation kit (Solaibao Biological Technology, #P8860) according to the manufacturer’s instructions. Freshly resected tumor tissues were digested and converted into a single cell suspension with the tumor dissociation kit (Miltenyi, #130-096-730). Blocking with anti-mouse CD16/32 (BioLegend, #01302) was conducted before staining immune cells with the specified antibody. Then surface staining was performed with antibodies including CD3, CD4, CD8, CD25, CD45, F4/80, CD11b and CD86 in the flow cytometry buffer (eBioscience™, #00-4222-57) and stained for 30 min on ice. FOXP3 staining was performed with a FOXP3/Transcription factor staining buffer set (eBioscience™, #00-5523-00) according to the manufacturer’s instructions. CD206 staining was performed immediately after cell surface antibody staining, followed by cell fixation using Fixation Buffer (BioLegend, #420801) for 30 min and permeabilization using Intracellular Staining PermeabilizationWash Buffer (BioLegend, #421002) for 15 min.

### Mitochondrial membrane potential measurement

The JC-1 staining according to the manufacture’s protocol (Beyotime, #C2006). In brief, cells were plated in 6-well plates at a density of 1 × 10^6^ cells/well. Following treatment, the cells were detached and suspended in medium containing with JC-1 staining buffer at 37 °C for 30 min in the dark, then subjected to flow cytometry analysis.

### Detection of reactive oxygen species

The intracellular levels of reactive oxygen species (ROS) were measured using an ROS assay kit (Beyotime, #S0033M) according to the manufacture’s protocol. Briefly, cells were harvested and incubated with 10 μM 2’,7’-Dichlorodihydrofluorescein diacetate (DCFH-DA) at 37 °C for 30 min, then washed three times with serum-free solution. The levels of ROS were quantified through flow cytometric analysis, followed by the measurement of the geometric mean fluorescence intensity (MFI).

### Transwell assays

For migration, 5 × 10^4^ cells were seeded into the upper chambers of transwell filters (Labselect). After a 24-h incubation, cells were fixed with 4% paraformaldehyde and stained with 1% crystal violet solution. Non-migrated cells inside the upper chamber were gently removed with cotton swabs, and the migrated cells at the bottom of the filter were photographed by counting three randomly microscopic fields per well and the mean was determined. For invasion, transwell plate was coated with diluted Matrigel matrix firstly, and the subsequent procedures were identical to those in the migration assays.

### Wound healing assay

Scratch wound assays were performed by quantifying the area of scratches at various time intervals. A total of 2 × 10^5^ cells were plated into each well of 6-well plates. After a 48-h incubation, a linear scratch was made with a 200 μL pipette tip at the bottom of the wells. Images of the same regions were captured at specified time points (0, 24 and 48 h) after wounding.

### ELISA assay

The ELISA kits (Shanghai Enzyme-linked, China) were performed for the detection of CSF2 levels in serum and in culture supernatant following the manufacturer’s protocols.

### CRC cells-macrophage cells coculture

A transwell insert with a 3.0 μm polycarbonate membrane pore size for 6-well plate (Labslect) was used for the coculture experiments. For the HCT116-macrophage cell coculture, M1 macrophages were induced by THP-1 cells as described previously. M1 macrophages were plated 48 h in a 6-well plate in advance and then cocultured with HTC116 at a 1:2 ratio at 37 °C. After 48 h, the macrophages were harvested, and the mean fluorescence intensity (MIF) of CD86 expression levels was analyzed. For the CT26-macrophage cell coculture, RAW264.7 macrophages were differentiated into M1 macrophages as described previously. RAW264.7 cells were plated 48 h in a 6-well plate in advance and then allowed interaction with CT26 at a 1:2 ratio at 37 °C. After 48 h, macrophages were collected and the CD86 expression levels were measured.

### Western blot

Total proteins from HCT116 and LoVo cells were lysed with RIPA buffer (Beyotime, #P0013B) which was supplemented with 50× Protease and phosphatase inhibitor cocktail (Beyotime, #P1008 and #P1045), in accordance with the provided protocol. An equal amount of total protein lysates (20 μg) was loaded onto SDS-PAGE gel and subsequently transferred to PVDF membranes (Millipore). After blocking with a diluted Protein Free Rapid Blocking solution (EpiZyme, #PS108P), the membranes were washed with TBST (Servicebio, #G2150) and incubated with primary antibodies at 4 °C overnight. The membranes were washed with TBST three times and then incubated with secondary antibodies for 1 h at room temperature. The antibodies were detailed in Table 1.

**Table 1:** Antibodies used in this study.

Protein name	Product number	Dilution ratio
PARP	CST, #9532	1:1000
BCL-2	Abcam, ab182858	1:1000
Cyclin B1	Abcam, ab32053	1:1000
CDK1	Abcam, ab201008	1:1000
JAK2	Immunoway, YT2426	1:1000
p-JAK2	Abmart, T56570	1:1000
STAT3	Abcam, ab68153	1:1000
p-STAT3	Abcam, ab76315	1:1000
PD-L1	Abmart, M033179	1:1000
Ki67	Proteintech, 280741-1-AP	1:200
CD86	CST, #91882	1:200
PE anti-mouse CD86	BioLegend, #159203	1 μg/Test
FITC anti-human CD86	BioLegend, #374203	1 μg/Test
FITC anti-mouse CD3	BioLegend, #100203	1 μg/Test
APC anti-mouse CD8a	BioLegend, #100711	1 μg/Test
PE anti-mouse CD4	BioLegend, #100407	1 μg/Test
APC anti-mouse CD25	eBioscience™, #17-0251-82	1 μg/Test
CD16/32	BioLegend, #101319	1 μg/Test
Brilliant violet 421 anti-mouse CD45	BioLegend, #103134	1 μg/Test
PE/Cy7 anti-mouse F4/80	BioLegend, #123113	1 μg/Test
FITC anti-mouse CD11b	BioLegend, #101205	1 μg/Test
APC anti-mouse CD206	BioLegend, #141707	1 μg/Test
PE anti-Mo/Rt Foxp3	eBioscience™, #12-5773-80	1 μg/Test
FITC anti-mouse CD4	eBioscience™, #11-0042-82	1 μg/Test
GAPDH	ABclonal, AC033	1:100,000
HRP* Goat Anti Mouse IgG	Immunoway, RS0001	1:10,000
HRP* Goat Anti Rabbit IgG	Immunoway, RS0002	1:10,000

### RNA isolation and real time PCR

Total RNA was extracted using the MolPure® Cell/Tissue Total RNA kit (Yeasen, #19211ES) and quantified with a NanoDrop-2000 spectrophotometer (Thermo Fisher). Reverse transcription was performed using the Hifair® II 1st Strand cDNA Synthesis Kit (gDNA digester plus) (Yeasen, #11123ES) according to the protocols, to synthesize cDNA. The amplification of the target genes was performed using 2× ChamQ SYBR qPCR Master Mix (Low Rox Premixed) (Vazyme, #Q331) on the QuantStudio5 real-time PCR system (Applied Biosystems). The specific primer sequences were shown in Table 2.

**Table 2:** Primers used in this study (5’-3’).

Gene symbol	Species	Forward (5’-3’)	Reverse (5’-3’)
ACTIN	Human	AAGTGTGACGTGGACATCCGC	CCGGACTCGTCATACTCCTGCT
CSF2	Human	TCCTGAACCTGAGTAGAGACAC	TGCTGCTTGTAGTGGCTGG
CD274	Human	TGGCATTTGCTGAACGCATTT	AGTGCAGCCAGGTCTAATTGT
CXCL8	Human	CACTGCGCCAACACAGAAAT	ATGAATTCTCAGCCCTCTTCAA
FGF19	Human	GCTGGAGATCAAGGCAGTCG	CGAGTACTGAAGCAGCCCCT
FOS	Human	TGGCGTTGTGAAGACCATGA	AGTTGGTCTGTCTCCGCTTG
JUN	Human	TCCAAGTGCCGAAAAAGGAAG	CGAGTTCTGAGCTTTCAAGGT
SESN2	Human	GAGCGGAACCTCAAGGTCTAT	GTTCACGTGGACCTTCTCTG
CHAC1	Human	GTGGTGACGCTCCTTGAAGA	AGGTAACCAGGGTTCTGCTC
DKK1	Human	TGACAACTACCAGCCGTACC	CAGGCGAGACAGATTTGCAC
CYP24A1	Human	CAGCCTGCTGCAGATTCTCT	CTTGTGGTACTCCACCAGGG
TUBB	Human	ATGTTCCTCGTGCCATCCTG	CTGCCCCAGACTGACCAAAT
INSIG1	Human	CCTGGCATCATCGCCTGTT	AGAGTGACATTCCTCTGGATCTG
NR2F1	Human	ATCGTGCTGTTCACGTCAGAC	TGGCTCCTCACGTACTCCTC
ALDH1A3	Human	ATCGACCTGGAGGGCTGTAT	ACGACGTTGTCATCTGTGGG
CD40	Human	ACTGAAACGGAATGCCTTCCT	CCTCACTCGTACAGTGCCA
CD80	Human	AAACTCGCATCTACTGGCAAA	GGTTCTTGTACTCGGGCCATA
IL-6	Human	ACTCACCTCTTCAGAACGAATTG	CCATCTTTGGAAGGTTCAGGTTG
IL-1β	Human	AGCTACGAATCTCCGACCAC	CGTTATCCCATGTGTCGAAGAA
CD163	Human	GCGGGAGAGTGGAAGTGAAAG	GTTACAAATCACAGAGACCGCT
CD206	Human	CAATTCCTGGCGATACCTCAG	GCACAACTCCGGTGACATCAA
IL-10	Human	GACTTTAAGGGTTACCTGGGTTG	TCACATGCGCCTTGATGTCTG
TGF-β	Human	CAATTCCTGGCGATACCTCAG	GCACAACTCCGGTGACATCAA
Gapdh	Mouse	AGGTCGGTGTGAACGGATTTG	TGTAGACCATGTAGTTGAGGTCA
Csf2	Mouse	GGCCTTGGAAGCATGTAGAGG	GGAGAACTCGTTAGAGACGACTT
IL-6	Mouse	TAGTCCTTCCTACCCCAATTTCC	TTGGTCCTTAGCCACTCCTTC
IL-1β	Mouse	GCAACTGTTCCTGAACTCAACT	ATCTTTTGGGGTCCGTCAACT
Cd40	Mouse	TGTCATCTGTGAAAAGGTGGTC	ACTGGAGCAGCGGTGTTATG
Cd80	Mouse	ACCCCCAACATAACTGAGTCT	TTCCAACCAAGAGAAGCGAGG
IL-6	Mouse	TAGTCCTTCCTACCCCAATTTCC	TTGGTCCTTAGCCACTCCTTC
IL-1β	Mouse	GCAACTGTTCCTGAACTCAACT	ATCTTTTGGGGTCCGTCAACT
TGF-α	Mouse	GAGTGACAAGCCTGTAGCC	CTCCTGGTATGAGATAGCAAA
Cd206	Mouse	CTCTGTTCAGCTATTGGACGC	TGGCACTCCCAAACATAATTTGA
IL-10	Mouse	GCTCTTACTGACTGGCATGAG	CGCAGCTCTAGGAGCATGTG
TGF-β	Mouse	CTCCCGTGGCTTCTAGTGC	GCCTTAGTTTGGACAGGATCTG
Arg1	Mouse	CTCCAAGCCAAAGTCCTTAGAG	AGGAGCTGTCATTAGGGACATC

### RNA sequencing and data analysis

Total RNA was extracted using the TRIzol reagent (Thermo Fisher, #15596018CN), following the manufacturer’s protocol, and then sent to OE Biotech Co., Ltd. (Shanghai, China). Subsequently, the libraries were constructed using VAHTS Universal V6 RNA-seq Library Prep Kit according to the manufacturer’s instructions. The quantified and validated libraries were sequenced on the Illumina Novaseq 6000 platform, generating 150 bp paired-end reads. The differentially expressed genes (DEGs) were identified using DESeq2, then underwent Kyoto Encyclopedia of Genes and Genomes (KEGG) pathway analysis, gene set enrichment analysis (GSEA) and Ingenuity pathway analysis (IPA).

### Hematoxylin eosin (H&E) and immunohistochemistry (IHC) staining analysis

For H&E staining, paraffin sections of tumor tissues were deparaffinized, rehydrated, and stained with hematoxylin and eosin according to a standard protocol. For IHC staining, slides of paraffin-embedded tumor tissues were dewaxed, antigen-retrieved, blocked by hydrogen peroxide. After blocking with 5% goat serum for 1 h, the anti-Ki67 antibody (1:200) and the anti-CD86 antibody (1:200) were added and incubated overnight at 4 °C. Afterwards, the secondary antibody was added and incubated at 37 °C for 30 min, followed by 100 μL of DAB working solution, and incubation at room temperature for 5 min.

### Statistical analysis

Statistical analysis was conducted using GraphPad Prism software (V.9.0). The results are presented as the means ± standard deviation of three independent experiments, and the data were analyzed using Student’s *t*-test to assess the significant difference between the two groups. *P* < 0.05 was considered statistically significant.

## Results

### NCTD efficiently inhibited tumor growth and promoted anti-tumor immunity in vivo

To assess the potential involvement of NCTD in vivo, we examined the effect of NCTD on the *balb/c* and the *balb/c* nude mice, respectively. In a mouse subcutaneous model constructed from CT26 cells, we found that NCTD treatment markedly reduced tumor volume and weight compared to the control group. The tumor growth was reduced by 23.3% ± 12.3% (*n* = 5, *P* < 0.05) and 54.8% ± 21.4% (*n* = 5, *P* < 0.01) in *balb/c* mice, at the NCTD doses of 2 mg/kg and 4 mg/kg, respectively (Fig. 1b–d). Similar results were observed in the subcutaneous tumor mouse model constructed from HCT116 cells. Specifically, tumor growth was diminished by approximately 33.3% ± 5.3% (*n* = 5, *P* < 0.01) and 51.3% ± 10.7% (*n* = 5, *P* < 0.001) in *balb/c* nude mice administered NCTD doses of 2 mg/kg and 4 mg/kg, respectively (Fig. 1f–h).

Tumor tissues were then subjected to H&E staining and IHC analysis. In the *balb/c* model, NCTD markedly reduced tumor cell density and differentiation in mice along with increased CD86 expression (Fig. 1e and Supplementary Fig. S1e). Similar to the results observed in the *balb/c* nude model, an elevation of CD86 expression was observed in tumor tissues of mice treated with NCTD (Fig. 1i). The changes of M1 macrophages which stained with F4/80, Cd11b and CD86 in tumor tissues, peripheral blood and spleen were detected (Fig. 1j, k). Interestingly, the M1 macrophages in tumor tissues, peripheral blood and spleen were remarkably increased in the NCTD-treated mice compared with the control mice. These results suggested that NCTD suppressed the growth of CRC cells and promoted M1 macrophage polarization in vivo.

To examine the effects of NCTD on antitumor immunity, we analyzed the percentages of CD4^+^ and CD8^+^ T cells in the peripheral blood and spleen. In peripheral blood, a significant increase in the percentage of CD8^+^ T cells and a reduction in the percentage of CD4^+^ T cells were observed in NCTD-treated mice compared to the control. However, the content of CD8^+^ and CD4^+^ T cells did not show significant changes in the spleen following NCTD treatment (Supplementary Fig. S1a, b). To further clarify the subtypes of CD4^+^ T cells, we examined the expression of CD25 and FOXP3 on CD4^+^ T cells to define Tregs. Compared with the control group, Treg cells in peripheral blood and spleen did not change markedly in the experimental groups (Supplementary Fig. S1c, d). These observations indicate that NCTD efficiently inhibited tumor growth and promoted M1 macrophage polarization in these mice with CRC tumors.

### NCTD promoted macrophages polarization to the M1 phenotype

**Fig. 2:**
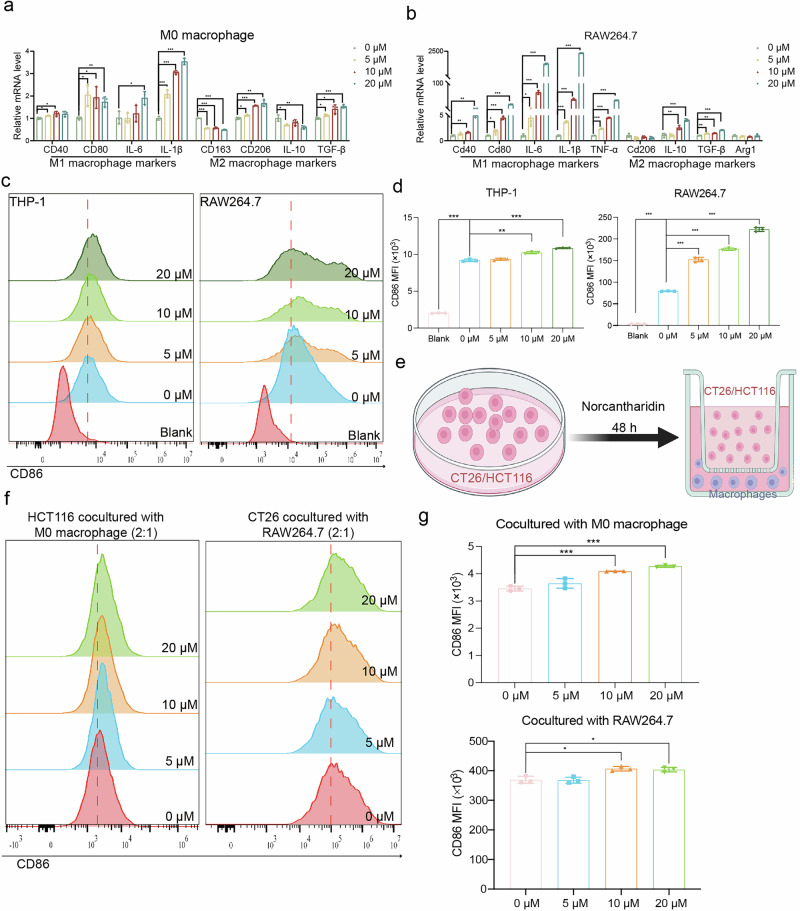
NCTD augments M1 macrophage polarization. **a, b** The mRNA expression levels of M1 and M2 macrophage markers in M0 (polarized THP-1) and RAW264.7 cells were measured by qPCR, respectively (*n* = 3). **c** Flow cytometry analysis of the expression of CD86 in THP-1 and RAW264.7 cells. **d** The mean fluorescence intensity of CD86 quantified from c (*n* = 3). **e** Schematic illustration of macrophages coculture with NCTD-treated CRC cells. **f** Flow cytometry analysis of the expression of CD86 in macrophages that cocultured with CRC cells. **g** The corresponding quantitative analysis of CD86 by flow cytometry (*n* = 3). Data are presented as mean ± SD. *P < 0.05, **P < 0.01, ***P < 0.001.

To confirm the observations above we investigated whether NCTD participates in M1 macrophage polarization in human monocytic THP-1 cell-derived macrophages and mouse macrophage RAW264.7 cells. The mRNA levels of classical markers for M1 and M2 macrophages were assessed by qPCR. As shown in Fig. 2a, b, NCTD treatment markedly upregulated the expression of M1 macrophage markers including CD40, CD80, IL-6, IL-1β, and TNF-α. However, no consistent trend was observed in the expression of M2 macrophage markers, including CD206, CD163, IL-10, TGF-β, and ARG1, in response to NCTD. The protein expression level of CD86 (a M1 macrophage marker) was further measured by flow cytometry in THP-1-derived and RAW264.7 macrophages (Fig. 2c). Quantitative analysis of the mean fluorescence intensity of staining for CD86 confirmed the remarkable upregulation of CD86 in THP-1 monocytes treated with LPS and IFN-γ, and in RAW264.7 macrophages treated with IFN-γ for 48 h, and this upregulation was promoted by NCTD in a concentration-dependent manner (Fig. 2d). As we all known, macrophage functional phenotype is shaped by proinflammatory or anti-inflammatory cytokines, and tumor cell-derived cytokines are important components of the TME. Next, we sought to evaluate whether NCTD could promotes M1 macrophage polarization through HCT116 and CT26 cells. We co-cultured CRC cells with macrophages at a 2:1 ratio (Fig. 2e). After 48 h of co-culture, the macrophage phenotype was analyzed by flow cytometry using the M1 macrophage surface marker CD86 (Fig. 2f). Flow cytometry showed that CD86 expression was increased on macrophages co-cultured with CRC cells treated with NCTD compared to those not exposed to NCTD (Fig. 2g). Taken together, these results strongly support that NCTD effectively promoted M1 macrophage polarization.

### NCTD inhibited cell viability and proliferation

**Fig. 3:**
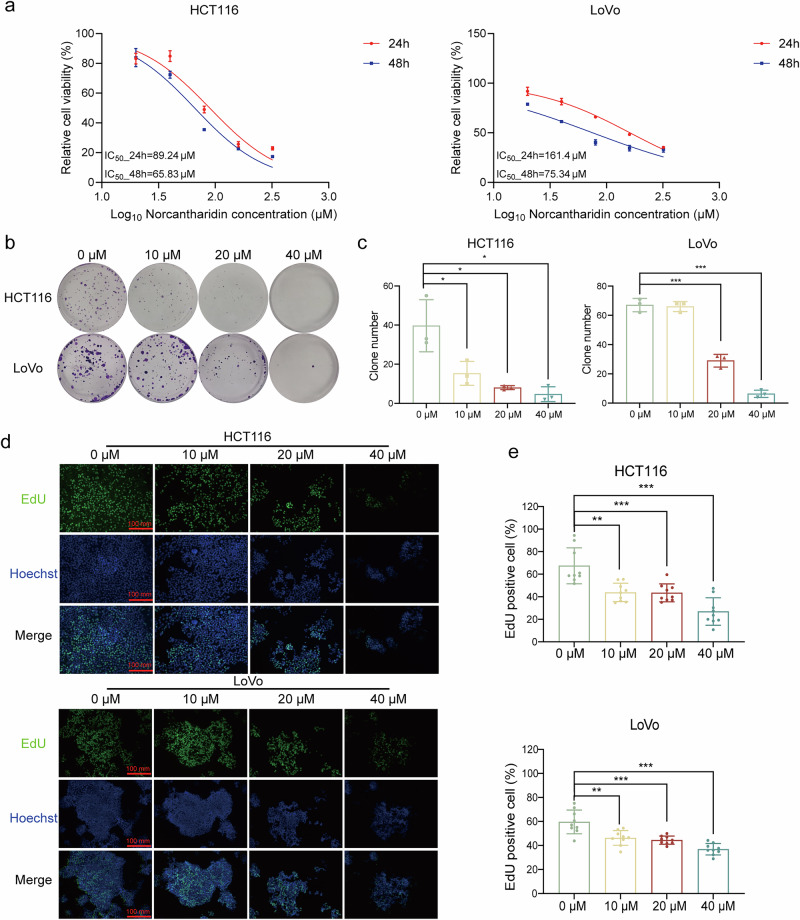
NCTD inhibits proliferation of CRC cells. **a** Cell viabilities of HCT116, LoVo cells after incubation with various concentrations of NCTD for 24 and 48 h (*n* = 3). **b** Cell proliferation was measured by colony formation in 12-well plates for 2 weeks with crystal violet staining. Representative photographs are shown. **c** The number of colonies was calculated using ImageJ (*n* = 3). **d** Representative fluorescence images of EdU staining (scale bar: 100 mm). **e** Statistical analysis of EdU positive cells was shown (*n* = 9). The results are presented as mean ± SD. *P < 0.05, **P < 0.01, ***P < 0.001.

To verify the reported cytotoxic effects of NCTD [6], CCK-8 assay was performed to detect its inhibitory effects on CRC cells. The results suggested that cell viability was significantly inhibited in a concentration- and time-dependent manner by NCTD, and the half-maximal inhibitory concentration (IC_50_) of NCTD on HCT116, LoVo was shown in Fig. 3a. Subsequent colony formation assay revealed that exposure to higher concentrations of NCTD resulted in a significant inhibition of proliferation in HCT116 and LoVo cells (Fig. 3b, c). As expected, the results of the EdU assay indicated the inhibition of cell proliferation by NCTD (Fig. 3d, e). These data suggested that NCTD can directly inhibit cell proliferation.

### NCTD attenuated CRC cell migration, invasion and arrested cell cycle

**Fig. 4:**
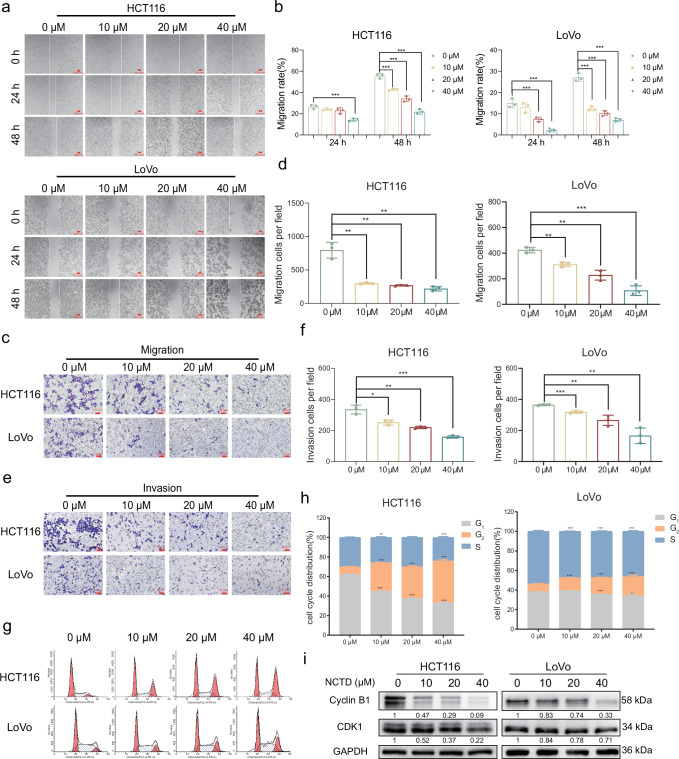
NCTD suppresses cell migration, invasion and arrests cell cycle. **a** Wound-healing experiments of HCT116 and LoVo cells for 24 h and 48 h (scale bar: 100 mm). **b** Cell migration rates were plotted by respective histogram (*n* = 3). **c, d** Representative images and cell count of migration assays for HCT116 and LoVo cells (scale bar: 50 μm, *n* = 3). **e, f** Representative images and cell count of invasion assays for HCT116 and LoVo cells (scale bar: 50 μm, *n* = 3). **g, h** Cell cycle of HCT116 and LoVo cells was determined by using flow cytometry (*n* = 3). **i** The expression of G_2_-related proteins (CDK1, Cyclin B1) was detected by Western blotting. The results are presented as mean ± SD. *P < 0.05, **P < 0.01, ***P < 0.001.

To further explore the role of NCTD in CRC cell migration and invasion, the wound-healing and transwell assays were employed to assess cellular migration in vitro. For the wound-healing assay, HCT116 and LoVo cells were incubated with various concentrations of NCTD for 0, 24 and 48 h, then photographed to calculate the scratch area. The results showed that NCTD resulted in a significant slower closing of scratch wounds compared with control (Fig. 4a, b). Based on these results, the transwell assay showed that NCTD decreased the numbers of migrating cells (Fig. 4c, d). In parallel, we observed that the aggressiveness of CRC was suppressed by NCTD in a cell invasion assay (Fig. 4e, f). Immediately afterwards, the cell cycle status was assessed using flow cytometry. The analysis revealed a notable increase in the proportion of cells in the G_2_ phase following NCTD administration (Fig. 4g, h). Consistent with the cell cycle analysis, Western blot analysis confirmed NCTD effectively reduced the expression of G_2_ phase-related proteins CDK1 and Cyclin B1 (Fig. 4i).

### NCTD induced apoptosis and ROS production in CRC cells

**Fig. 5:**
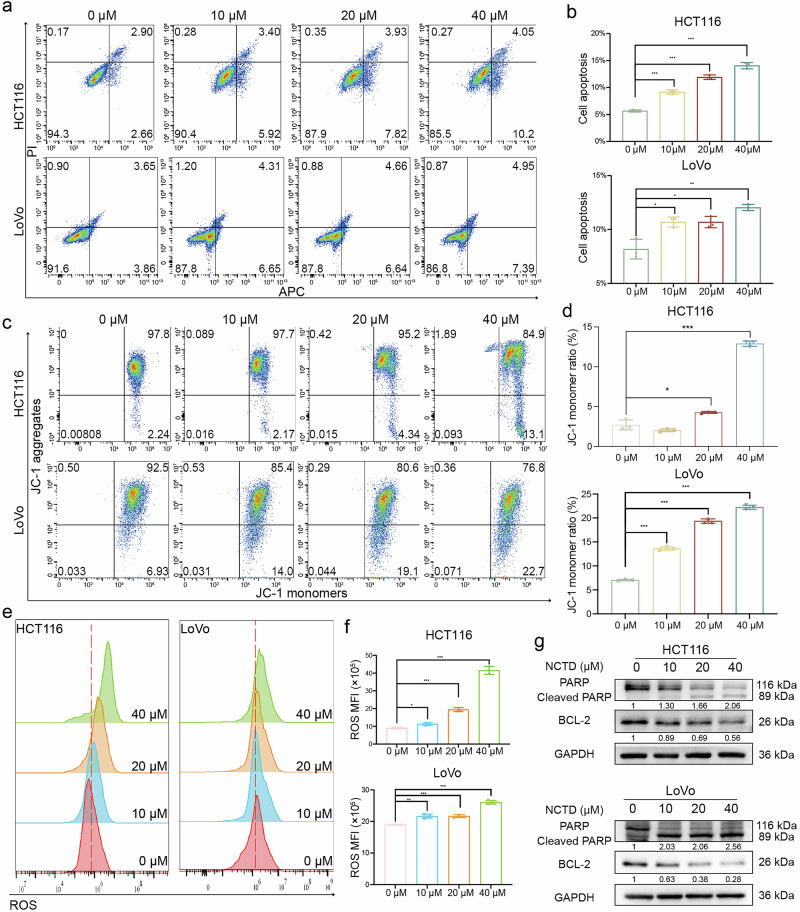
NCTD induces apoptosis and ROS in CRC cells. **a** The representative images of cell apoptosis in HCT116 and LoVo cells for 48 h, respectively. **b** The statistical results of cell apoptosis assays (*n* = 3). **c** Flow cytometry analysis of mitochondrial membrane potential levels of HCT116 and LoVo cells stained with JC-1. **d** Statistical analysis of the red/green fluorescence signal ratio of JC-1 (*n* = 3). **e** ROS levels assessed by flow cytometry measuring DCFH-DA fluorescence in HCT116 and LoVo cells for 48 h. **f** Quantitative analysis of mean fluorescence intensity for ROS was shown (*n* = 3). **g** The expression of apoptosis markers (PARP, BCL-2) was detected by Western blotting. Data are presented as mean ± SD. *P < 0.05, **P < 0.01, ***P < 0.001.

Given that cell cycle arrest and induction of apoptosis are two essential factors for inhibiting cell proliferation, Annexin V-APC/PI staining was performed to determine the apoptotic rate. As shown in Fig. 5a, b, NCTD resulted in a dose-dependent increase in early and late apoptosis in HCT116 and LoVo cells. Furthermore, JC-1 staining assays demonstrated a dysfunction in mitochondrial membrane potential in CRC cells by NCTD in a concentration-dependent manner (Fig. 5c, d). To clarify whether NCTD-induced apoptosis was due to ROS production, ROS assay was applied to detect ROS level in HCT116 and LoVo cells. As shown in Fig. 5e, f, NCTD resulted in a dose-responsive increase in intracellular ROS levels in CRC cells. Western blot analysis revealed that the anti-apoptotic protein BCL-2 was decreased, while cleaved PARP was significantly increased after 48 h of NCTD exposure (Fig. 5g).

### NCTD inhibited JAK2/STAT3 pathway and promoted CSF2 secretion

**Fig. 6:**
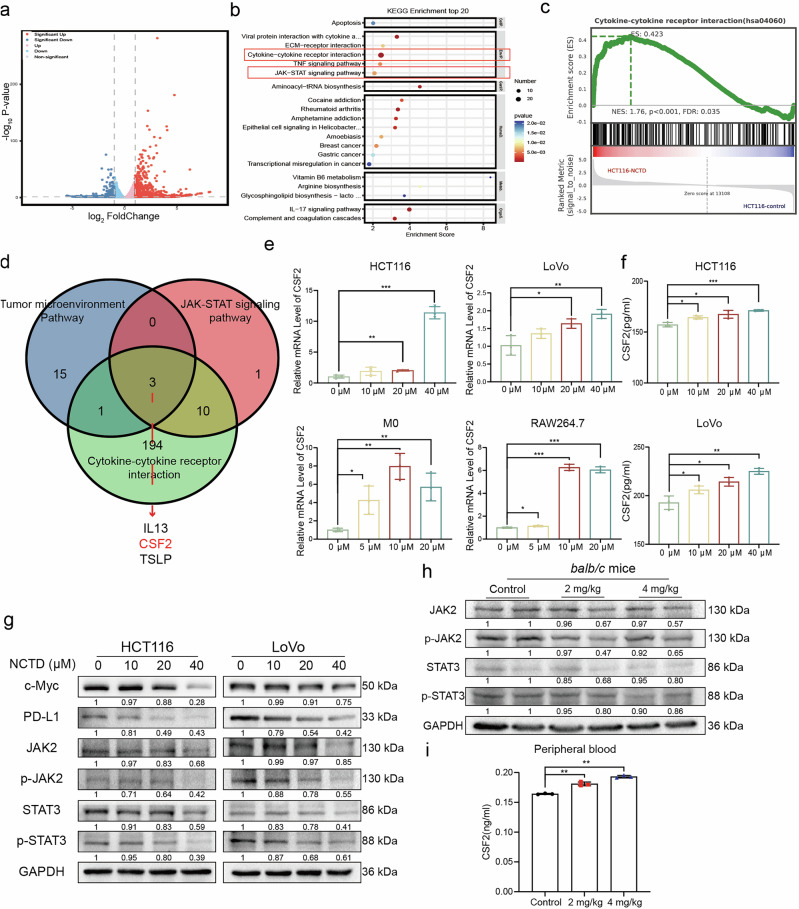
Transcriptome analysis and validation of NCTD-regulated differentially expressed genes. **a** Volcano plot for differentially expressed genes (DEGs): fold change ≥ 2, *P* < 0.05. **b** KEGG analysis for DEGs. **c** Enrichment results for cytokine-cytokine receptor interaction via GSEA. **d** Venn diagram showing the number of genes expressed in significantly enriched pathways. **e** qPCR analysis of CSF2 mRNA levels in HCT116, LoVo, M0 and RAW264.7 cells. **f** The levels of CSF2 in the culture medium of HCT116, LoVo cells as determined by ELISA assays (*n* = 3). **g** Expression levels of JAK2/STAT3 signaling pathway related proteins as determined by Western blotting. **h** The protein levels of JAK2, STAT3, p-JAK2 and p-STAT3 in CRC tissues measured by Western blot**. i** Levels of CSF2 in peripheral blood were detected by ELISA kits (*n* = 3). Data are presented as mean ± SD. *P < 0.05, **P < 0.01, ***P < 0.001.

To further explore the molecular response induced by NCTD, we conducted RNA-sequencing to analyze the transcriptomes of PBS- and NCTD-treated HCT116 cells for 48 h. A volcano plot illustrating the differential expression of these genes was  shown in Fig. 6a. qPCR analysis confirmed the expression of selected differentially expressed genes confirming the reliability of RNA-sequencing data (Supplementary Fig. S2a, b). The genes that were expressed differently were analyzed using KEGG pathway enrichment analysis, which showed significant enrichment of the JAK-STAT signaling pathway, in line with previous findings [21]. Meanwhile, the cytokine-cytokine receptor interaction pathway was significantly enriched (Fig. 6b). Similarly, gene set enrichment analysis (GSEA) showed that the cytokine-cytokine receptor interaction pathway was significantly up-regulated (Fig. 6c). In addition, we performed pathway analysis of the differentially expressed genes using IPA, and the results showed prominent activation of the tumor microenvironment pathway (Supplementary Fig. S3). To further screen potential downstream candidates, we employed three different bioinformatic analyses to identify common gene candidates, as shown in the Venn diagram (Fig. 6d). CSF2 has attracted our attention due to its enriched expression in various signaling pathways and its regulation of TME [10]. Next, a dose-dependent increase in CSF2 induced by NCTD in CRC and macrophage cells, as well as in culture supernatants was observed, using qPCR and ELISA, respectively (Fig. 6e, f). To verify the enrichment of JAK/STAT3 pathway, the key proteins in the signaling pathway were measured by Western blot. The results indicated that NCTD significantly downregulated the expression of p-STAT3 (Tyr705) and p-JAK2 (Tyr1007/1008) in HCT116 and LoVo cells. Moreover, the downstream proteins of the STAT3 pathway, PD-L1 and c-Myc, were suppressed after 48 h of treatment (Fig. 6g). Meanwhile, Western blot confirmed the inhibitory effects of NCTD on p-STAT3 and p-JAK2 protein levels in tumor tissues (Fig. 6h). Additionally, ELISA analysis demonstrated significantly elevated CSF2 expression in the peripheral blood of the NCTD treated mice versus the control (Fig. 6i). These results imply that NCTD exerts an inhibitory effect on STAT3 and contributes to the CSF2-secreting phenotype.

### The antitumor effect of NCTD on CRC largely depended on macrophages

**Fig. 7:**
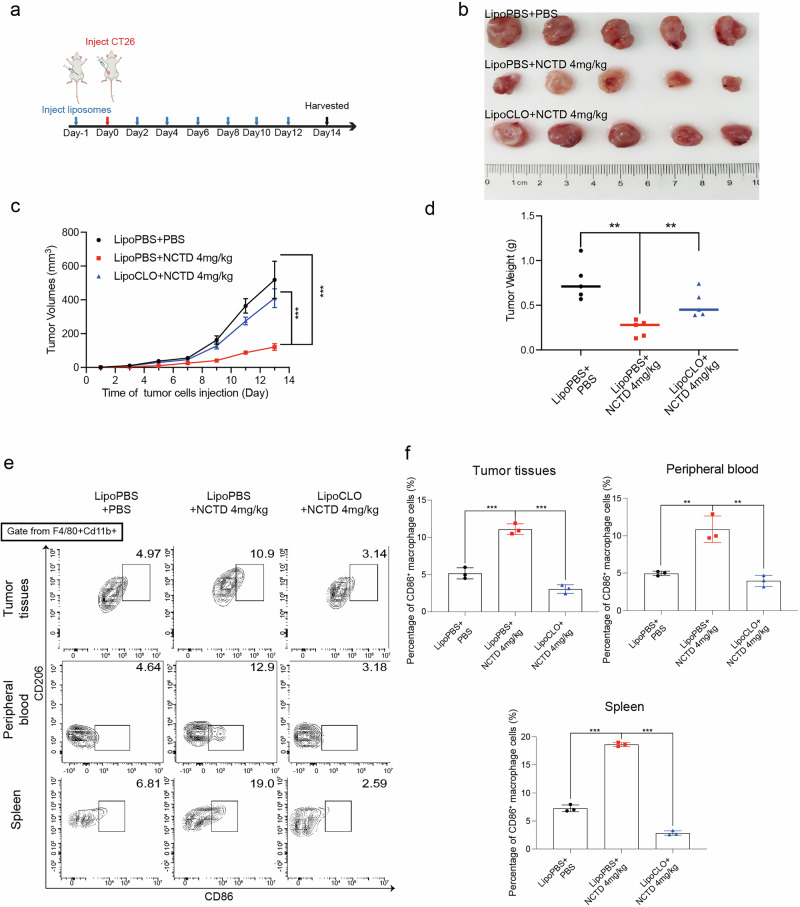
Antitumor effects of NCTD in macrophage clearance mouse model. **a** Schematic illustration of the treatment process of animal experiments. **b** Representative tumor photographs (*n* = 5). **c** The curves of changes in tumor volume (*n* = 5). **d** The average tumor weight (*n* = 5). **e** Results of flow cytometry analysis to detect the proportion changes of M1 and M2 macrophages in tumor tissues, peripheral blood and spleen of mice. **f** Statistical analysis of the percentages of CD86^+^ macrophage cells (*n* = 3). Data are presented as mean ± SD. **P < 0.01, ***P < 0.001.

As shown above, although NCTD demonstrated antitumor effects by promoting M1 polarization and enhancing the cytotoxicity of CRC cells, its specific role in NCTD-mediated therapy for CRC requires further investigation. To address this, a macrophage clearance mouse model was established using intraperitoneal injections of clodronate liposomes (Fig. 7a). The results showed that the group of NCTD and LipoPBS exhibited better antitumor effect than the LipoPBS group. However, in macrophage-depleted mice, the antitumor effects of NCTD were significantly diminished, as evidenced by comparisons of tumor volume and weight between the NCTD plus LipoPBS group and the NCTD plus LipoCLO group (Fig. 7b–d). Consistent with previous results, flow cytometry consistently showed that NCTD can induce the percentages of M1 macrophages in tumor tissues, peripheral blood and spleen. In contrast, the percentage of M1 macrophages in these tissues was significantly reduced in the NCTD plus LipoCLO group compared to the NCTD plus LipoPBS group (Fig. 7e, f). Taken together, these results suggest that macrophages are the primary effectors responsible for the antitumor activity of NCTD.

## Discussion

CRC is characterized by low immunogenicity and significant heterogeneity and presents a complex TME marked by immunosuppressive and inflammatory conditions, rendering it highly resistant to immunotherapy [22, 23]. Due to its low toxicity and significant antitumor effects, NCTD is viewed as a routine anticancer agent. It has been reported that NCTD on inhibition of hepatocellular carcinoma through miR-214 modulating macrophage polarization [19]. Nevertheless, our results differ significantly from this study in the hepatocellular cancer in the following aspects. First, our study focuses on the role of NCTD in colorectal cancer, not hepatocellular cancer. Second, we used two macrophage models, mouse macrophage RAW264.7 and human mononuclear leukocyte THP-1 cells, to elucidate the role and mechanism of NCTD in promoting macrophage M1 polarization, while the previous study used mouse macrophage RAW264.7 cells or tumor-associated macrophages. Third, we performed additional experiment with the macrophage-clearance mouse model and further confirmed macrophages as the major effectors responsible for the antitumor effects of NCTD. Fourth, we found that NCTD promoted macrophage M1 polarization not only directly through increasing CSF2 but also by inducing colorectal cancer cells to secrete CSF2. In the present study, we investigated the anti-tumor effects of NCTD against CRC and uncovered the underlying mechanisms in the light of the M1 macrophage polarization, apoptosis and cell cycle arrest. We first observed the inhibitory effects of NCTD on the tumor growth in two subcutaneous CRC models. We also observed the infiltration of M1 macrophages in tumor tissues by immunohistochemistry, and detected the percentages of CD4, CD8 and other T cells subtypes in the peripheral blood and spleen tissues by flow cytometry. Next, we found that NCTD promoted macrophage M1 polarization using qPCR, flow cytometry and co-culture models in vitro. In addition, the inhibitory effects of NCTD on CRC cell proliferation, invasion, migration and inducing apoptosis of tumor cells were observed in vitro by CCK8, EdU staining, apoptosis and cycle analysis, wound healing and transwell assays. RNA sequencing of HCT116 cells incubated with NCTD revealed JAK2/STAT3 signaling pathway and CSF2 as the differentially expressed genes when compared with the control group and analyzed via multiple enrichment pathways. Further Western blot analysis confirmed that NCTD significantly inhibited JAK2/STAT3 signaling pathway. The effect of NCTD induced CSF2 secretion was further confirmed by qPCR and ELISA, leading to M1 macrophage polarization. Therefore, our findings provided compelling evidence that (1) NCTD induced M1 macrophage polarization and had a convincing anti-CRC effect both in vivo and in vitro; (2) NCTD promoted the accumulation of CSF2 secreted by CRC cells and macrophages and inhibited the JAK2/STAT3 pathway to induce apoptosis and suppresses proliferation, invasion and migration in CRC cells. These findings strongly support that NCTD possesses the capacity to improve immunosuppression and inflammatory TME and may serve as a new effective therapeutic drug for CRC.

An increasing number of studies have demonstrated that macrophages are highly dynamic and plastic cells that shape the TME [24], and reorienting and reshaping macrophages have been considered therapeutic strategies [25, 26]. Notably, several studies have reported that certain toxic medicines are crucial in cancer treatment by directly killing tumor cells and regulating macrophage polarization in the TME [27, 28]. Our results showed that NCTD increased CD86 levels within tumor tissues of NCTD-treated mice and promoted macrophage polarization to the M1 phenotype in vitro. Likewise, we observed that macrophages co-cultured with NCTD-treated CRC cells increased the propensity of M1 macrophage polarization. Furthermore, the macrophage clearance mouse model was constructed to elucidate the importance of macrophages in the antitumor effects exerted by NCTD in vivo. Our results showed that clearance of macrophages significantly reversed the antitumor effects of NCTD. Macrophages are the most abundant cells within TME and play a vital role in tumor initiation and progression [29]. In the context of tumor, they are divided into two phenotypes: the tumor-inhibiting M1 type and the tumor-promoting M2 type [6, 7]. M1 macrophages are associated with antitumor activity because they produce pro-inflammatory cytokines and promote immune responses against tumors. Accumulating researches have demonstrated that the important regulatory role of STAT3 signaling pathway in macrophage differentiation. Overall, inhibition of the STAT3 signaling pathway promotes macrophage M1 polarization and inhibits macrophage M2 polarization, and promotion of the STAT3 signaling pathway inhibits macrophage M1 polarization and promotes macrophage M2 polarization [30–33]. Our results indicated that NCTD exerts its anti-tumor effects by promoting the polarization of M1 macrophages, rather than M2 macrophages.

A previous study reported that NCTD possesses significantly stronger antitumor activity and lower toxicity [34]. In our study, NCTD exhibited a powerful anti-CRC activity. Flow cytometry results further confirmed that the number of apoptosis was significantly reduced after treatment with NCTD. In addition, we observed a concentration-dependent increase in cleaved PARP protein levels and a concentration-dependent decrease in BCL-2 protein levels in NCTD-treated CRC cells, suggesting that the cytotoxic effects of NCTD on CRC were associated with both the extrinsic and intrinsic apoptotic pathways. As expected, NCTD increases the production of ROS, leading to mitochondrial membrane potential dysfunction. The increase in ROS production by NCTD is associated with a decrease in mitochondrial membrane potential, which subsequently leads to mitochondrial dysfunction. This dysfunction is characterized by increased mitochondrial membrane permeability and the release of cytochrome *c* from mitochondria into the cytoplasm, promoting apoptosis in cancer cells [35]. Nevertheless, based on our observation that NCTD can cause ROS production in CRC cells, we cannot completely exclude a mechanism of ROS-induced M1 macrophages [36, 37].

Cell death involves not only apoptosis but also cell-cycle disruption. Formation of CDK1-cyclin B1 complexes is crucial for regulating G_2_/M progression [38]. Previous studies have shown that blocking CDK1-cyclin B1 complexes results in cell-cycle arrest at the G_2_/M phase [39, 40]. Our findings demonstrated that NCTD prompted a concentration-dependent accumulation of cells in the G_2_/M phase. This observation was further substantiated by the reduced expression levels of G_2_/M phase-related proteins, including CDK1 and cyclin B1.

To elucidate the potential mechanisms by which NCTD inhibits the development and progression of CRC, we performed RNA sequencing analysis. Subsequently, multiple enrichment analyses revealed that multiple genes in which the most remarkable results referred to CSF2, IL13 and Thymic stromal lymphopoietin (TSLP) (Fig. 6d). TSLP has been reported mainly in infectious and inflammatory diseases, whereas its role in tumors was mainly antitumor through T cell and dendritic cell activation [41, 42]. IL-13 is an immunomodulatory cytokine, which is more conducive to M2 polarization in macrophages [43], and inhibition of its secretion can exert antitumor effects [44]. It has been reported that CSF2, as a classical inflammatory inducer of macrophages, promotes their polarization to the M1 phenotype [10]. Our screening results and preliminary experimental data found that NCTD promoted significantly higher expression levels of CSF2 compared to IL-13 and TSLP, respectively. The primary objective of this study is to elucidate the novel core mechanisms of NCTD induced M1 polarization, with CSF2 demonstrating stronger experimental impact and clearer mechanistic relevance in driving this progress. Combined with related literature studies, this justifies prioritizing CSF2 over the other less relevant molecules. Furthermore, experimental findings suggested that NCTD promotes M1 macrophage polarization, with CSF2 likely serving as a potential downstream molecule. qPCR and ELISA assays were used to detect the expression of CSF2 in CRC and macrophage cells and their culture supernatants, as well as the peripheral blood of mice, suggesting that NCTD promoted CSF2 secretion. The JAK2/STAT3 pathway is pivotal in transmitting information from extracellular chemical signals to the cell nucleus, resulting in DNA transcription and subsequent cellular responses [45]. In the context of cancer, the JAK2/STAT3 signaling pathway is implicated in cell proliferation and survival [46]. It has been shown to mediate inflammatory responses by regulating the production of pro-inflammatory cytokines such as IL-6, IL-1β, and TNF-α [47]. Previous studies have noted that activation of STAT3 can promote macrophage polarization towards the M2 phenotype [48, 49]. Activated STAT3 forms dimers, translocates to the nucleus, and binds to specific promoter regions of target genes that control proliferation (such as c-Myc, Cyclin-B1, and BCL-2) and PD-L1, leading to their transcriptional activation [50]. Consisent with these findings, Western blot analyses in CRC tissues and cells demonstrated that NCTD suppressed phosphorylated JAK2 and STAT3 levels both in vivo and in vitro, confirming JAK2/STAT3 signaling as the mechanistic pathway underlying its antitumor activity (Fig. 6g, h). In our study, RNA sequencing analysis showed that enrichment of JAK-STAT pathways after NCTD-treatment. Along with Western blot validation, NCTD markedly downregulated the expression of p-STAT3 (Tyr705) and p-JAK2, meanwhile suppressed the levels of c-Myc and PD-L1 proteins, suggesting that NCTD suppressed JAK2/STAT3 signaling in CRC cells. Additionally, the treatment of HCT116 cells with a combination of an agonist of STAT3 (Glycochenodeoxycholic acid) with NCTD significantly reduced the expression of phosphorylated JAK2 and phosphorylated STAT3 compared to NCTD alone. However, NCTD plus a STAT3 inhibitor (Stattic) failed to significantly reduce phosphorylated JAK2 and STAT3 levels compared to NCTD alone (Supplementary Fig. S4). The tumor immunosuppressive microenvironment is mainly induced by the suppression of immune checkpoints. Previous studies have shown that compounds combined with PD-1 antibody for tumor treatment can significantly enhance the anti-tumor effect [28, 51]. In the present study, we found that NCTD reduced PD-L1 expression level by inhibiting JAK2/STAT3 signaling (Fig. 6g). Therefore, NCTD combined with PD-1 therapy is quite possible to enhance the antitumor effect synergistically.

**Fig. 8:**
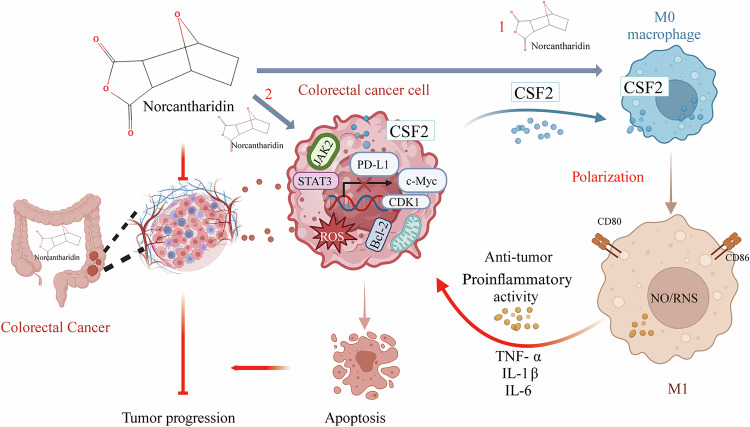
The illustration of the mechanisms for NCTD anticancer effects on colorectal cancer. Mechanism 1: NCTD stimulates to secretion of CSF2 from M0 macrophages and colorectal cancer cells to cause macrophage polarization toward a pro-inflammatory M1 phenotype. These activated M1 macrophages secrete anti-tumor cytokines (TNF-α, IL-1β, IL-6 etc.) to exert anti-tumor effects. Mechanism 2: Concurrently, NCTD directly inhibits JAK2/STAT3 phosphorylation in colorectal cancer cells, leading to downstream suppression of Bcl-2, c-myc, CDK1 and PD-L1, which ultimately induces apoptosis of CRC cells. These coordinated dual mechanisms highlight the multimodal anti-tumor action of NCTD on colorectal cancer.

In summary, this study demonstrated that NCTD inhibited the JAK2/STAT3 pathway in CRC cells and caused apoptosis of CRC cells and thus tumor suppression (mechanism 2 in Fig. 8). NCTD promoted macrophage M1 polarization mainly through inducing CSF2 secretion from both macrophages (mechanism 1 in Fig. 8) and CRC cells (mechanism 2 in Fig. 8). However, future additional experimental validation of CSF2 and its regulation, as well as proper exclusion of IL-13 and TSLP in the effects of NCTD on CRC or other types of tumor cells may provide further insights into the particular anticancer mechanism of NCTD.

## Supplementary information

Figure S1

Figure S2

Figure S3

Figure S4

Supplementary information
