# Supplementary figures and images for: Norcantharidin promotes M1 macrophage polarization and suppresses colorectal cancer growth
Source: Acta Pharmacol Sin. 2025 May 20;46(10):2820–34. doi: 10.1038/s41401-025-01578-8 (PMC12460879; doi:10.1038/s41401-025-01578-8)

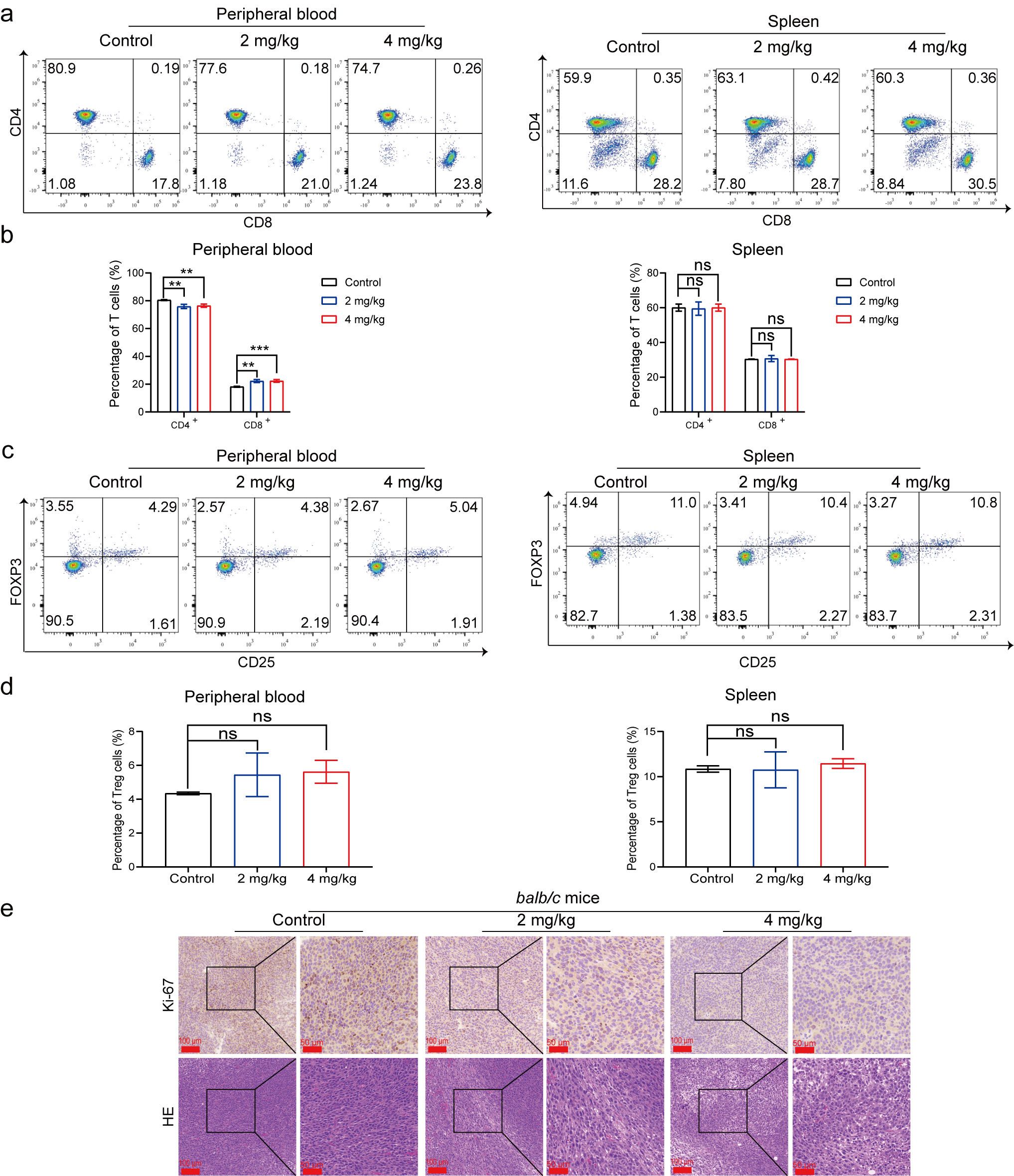

Supplement: Supplementary file 1 — Figure S1 [file 41401_2025_1578_MOESM1_ESM.tif]

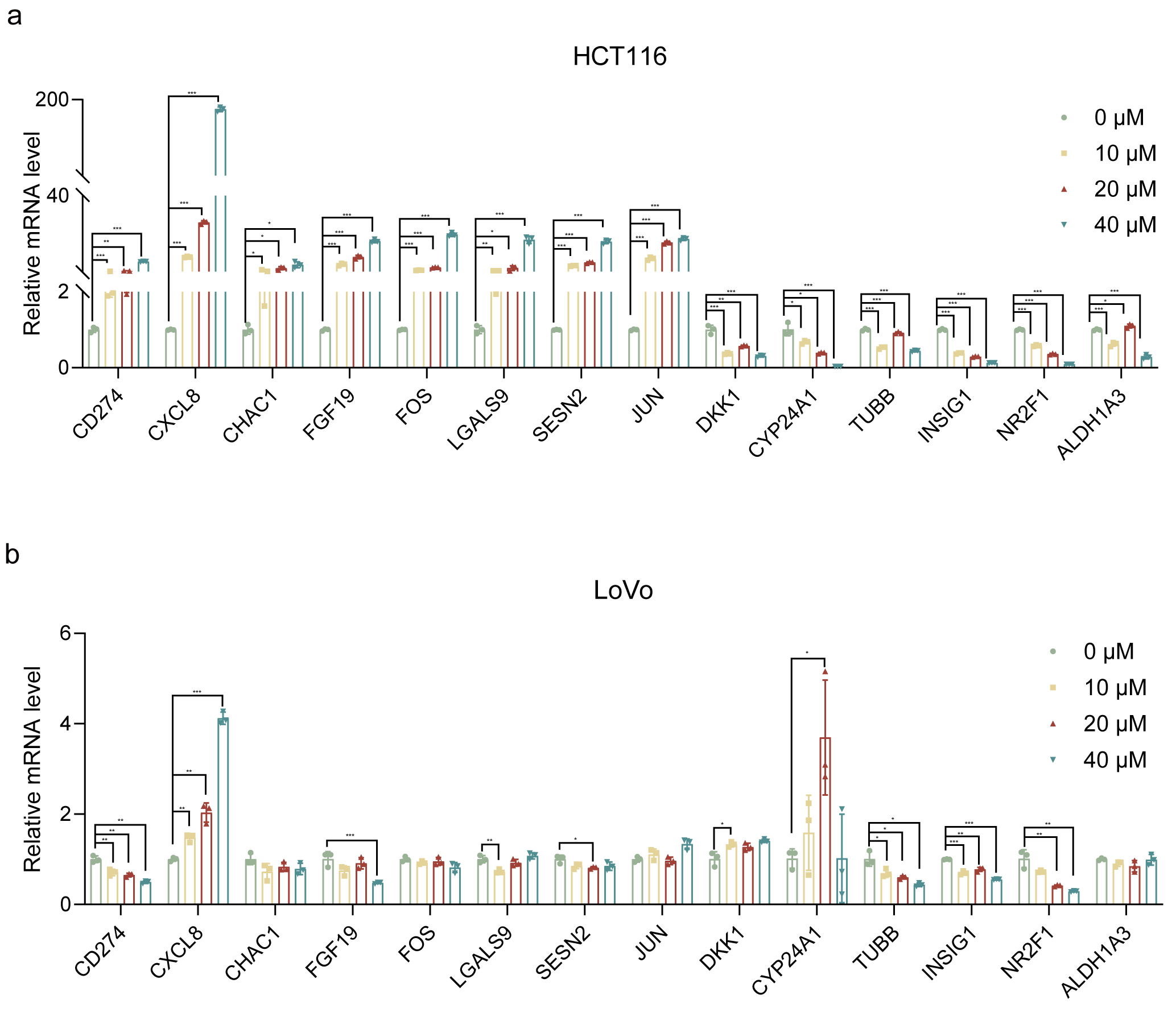

Supplement: Supplementary file 2 — Figure S2 [file 41401_2025_1578_MOESM2_ESM.tif]

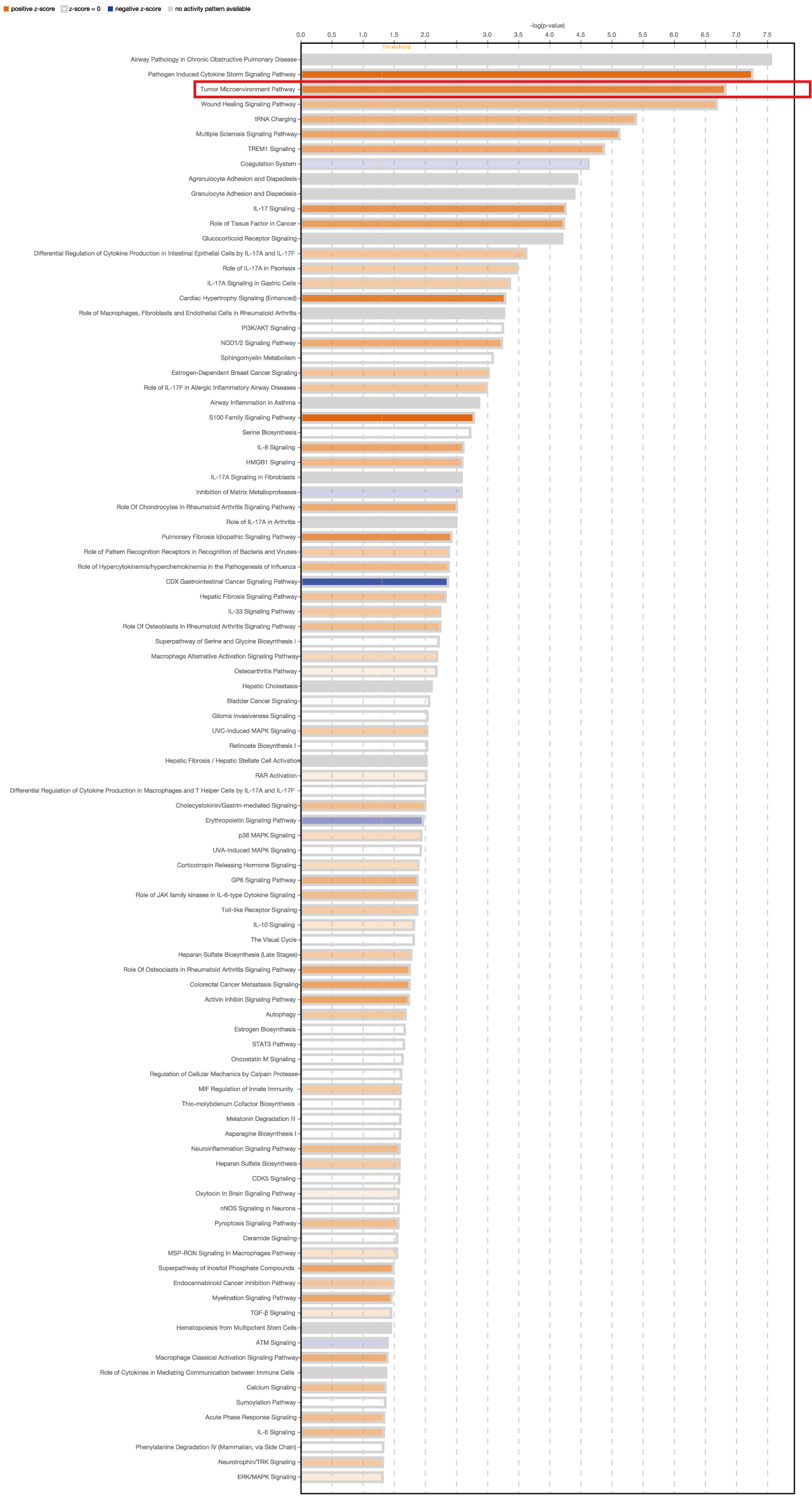

Supplement: Supplementary file 3 — Figure S3 [file 41401_2025_1578_MOESM3_ESM.tif]

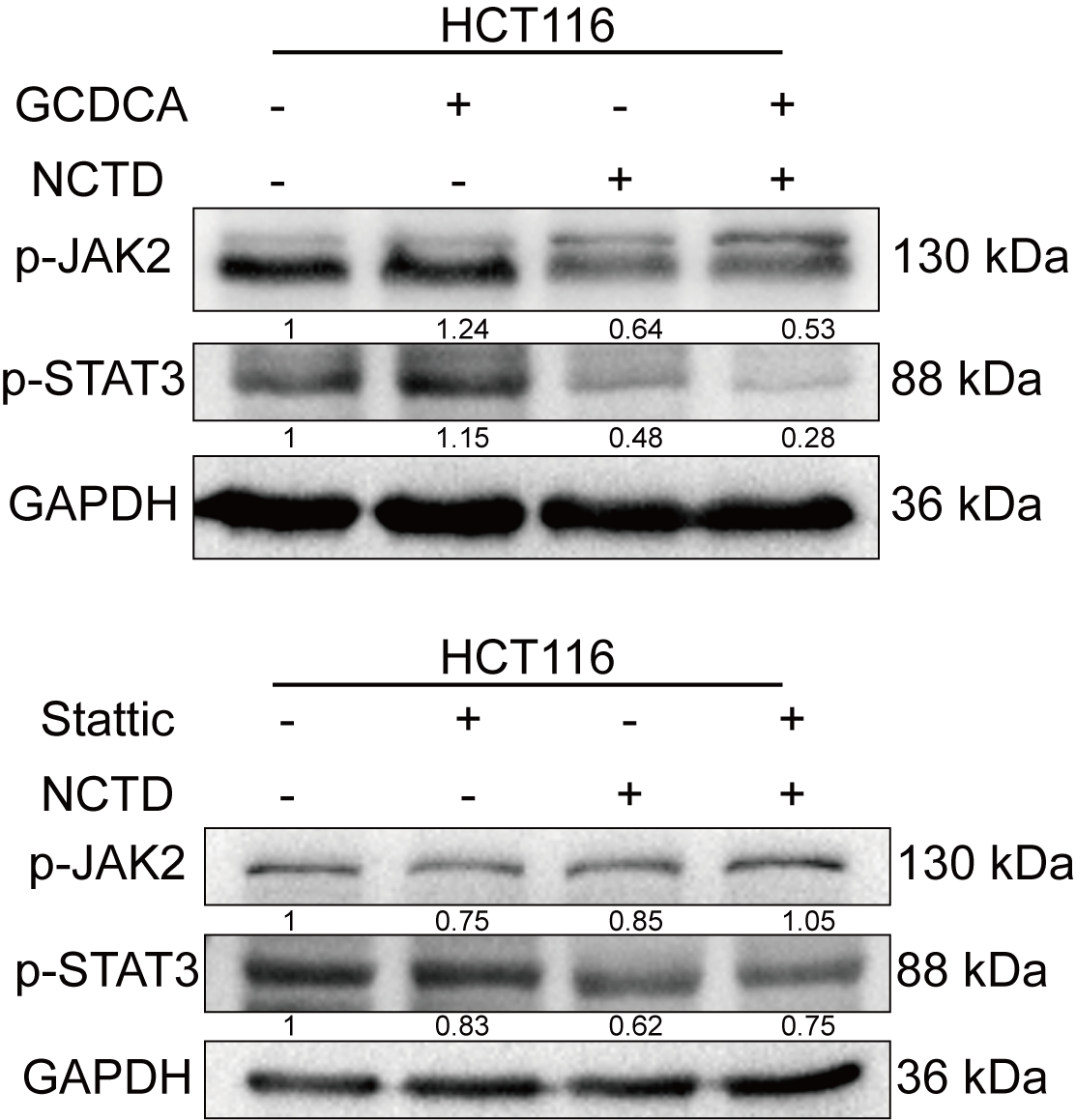

Supplement: Supplementary file 4 — Figure S4 [file 41401_2025_1578_MOESM4_ESM.tif]
