# Supplementary material for: Norcantharidin promotes M1 macrophage polarization and suppresses colorectal cancer growth
Source: Acta Pharmacol Sin. 2025 May 20;46(10):2820–34. doi: 10.1038/s41401-025-01578-8 (PMC12460879; doi:10.1038/s41401-025-01578-8)
Supplement: Supplementary file 5 — Supplementary information [file 41401_2025_1578_MOESM5_ESM.docx]

**Fig. S1 Evaluation of NCTD on tumor immune microenvironment using flow cytometry and IHC and H&E staining.** **(a, b)** The percentage of CD4^+^ and CD8^+^ T cells in the peripheral blood and spleen (*n*=3). **(c, d)** The percentage of Treg cells in the spleen (*n*=3). **(e)** Representative H&E staining, Ki-67 immunohistochemistry images of each group of tumors (200× magnification, scale bar: 50 μm). Data are presented as mean ± SD. ns *P*>0.05, ***P* < 0.01, ****P* < 0.001.

**Fig. S2 Verification of transcriptomic sequencing results by qPCR.** **(a)** The 14 differentially expressed genes were verified by qPCR in HCT116 cell. **(b)** The 14 differentially expressed genes were verified by qPCR in LoVo cell. Data are presented as mean ± SD. **P* < 0.05, ***P* < 0.01, ****P* < 0.001.

**Fig. S3 Ingenuity Pathway Analysis (IPA) analysis of the differentially expressed proteins in the NCTD and control group.**

**Fig. S4 The levels of p-JAK2 and p-STAT3 were examined using Western blotting in HCT116 cells stimulated with NCTD (40 μM), STAT3 agonist (Glycochenodeoxycholic acid, 250 μM) and STAT3 inhibitor (Stattic, 5 μM).**
